# Andrographolide Inhibits Nuclear Factor-**κ**B Activation through JNK-Akt-p65 Signaling Cascade in Tumor Necrosis Factor-*α*-Stimulated Vascular Smooth Muscle Cells

**DOI:** 10.1155/2014/130381

**Published:** 2014-07-10

**Authors:** Yu-Ying Chen, Ming-Jen Hsu, Cheng-Ying Hsieh, Lin-Wen Lee, Zhih-Cherng Chen, Joen-Rong Sheu

**Affiliations:** ^1^Graduate Institute of Medical Sciences, College of Medicine, Taipei Medical University, Taipei 11031, Taiwan; ^2^Department of Pharmacology, School of Medicine, Taipei Medical University, Taipei 11031, Taiwan; ^3^Department of Microbiology and Immunology, Taipei Medical University, Taipei 11031, Taiwan; ^4^Department of Cardiology, Chi-Mei Medical Center, Tainan City 71004, Taiwan; ^5^Department of Pharmacy, Chia Nan University of Pharmacy & Science, Tainan City 71004, Taiwan

## Abstract

Critical vascular inflammation leads to vascular dysfunction and cardiovascular diseases, including abdominal aortic aneurysms, hypertension, and atherosclerosis. Andrographolide is the most active and critical constituent isolated from the leaves of *Andrographis paniculata*, a herbal medicine widely used for treating anti-inflammation in Asia. In this study, we investigated the mechanisms of the inhibitory effects of andrographolide in vascular smooth muscle cells (VSMCs) exposed to a proinflammatory stimulus, tumor necrosis factor-*α* (TNF-*α*). Treating TNF-*α*-stimulated VSMCs with andrographolide suppressed the expression of inducible nitric oxide synthase in a concentration-dependent manner. A reduction in TNF-*α*-induced c-Jun N-terminal kinase (JNK), Akt, and p65 phosphorylation was observed in andrographolide-treated VSMCs. However, andrographolide affected neither I*κ*B*α* degradation nor p38 mitogen-activated protein kinase or extracellular signal-regulated kinase 1/2 phosphorylation under these conditions. Both treatment with LY294002, a phosphatidylinositol 3-kinase/Akt inhibitor, and treatment with SP600125, a JNK inhibitor, markedly reversed the andrographolide-mediated inhibition of p65 phosphorylation. In addition, LY294002 and SP600125 both diminished Akt phosphorylation, whereas LY294002 had no effects on JNK phosphorylation. These results collectively suggest that therapeutic interventions using andrographolide can benefit the treatment of vascular inflammatory diseases, and andrographolide-mediated inhibition of NF-*κ*B activity in TNF-*α*-stimulated VSMCs occurs through the JNK-Akt-p65 signaling cascade, an I*κ*B*α*-independent mechanism.

## 1. Introduction

Coronary artery disease (CAD) represents the leading cause of mortality and morbidity in developed countries, and atherosclerosis is the hallmark of many critical events in the pathogenesis of CAD [1]. Consequently, developing novel therapeutic agents for atherosclerosis patients is a major research priority. One of the risk factors for atherosclerosis is chronic and mild inflammation of the arteries [2]. Therefore, the inhibition of vascular smooth muscle cell (VSMC) inflammation might be a major target for the treatment of cardiovascular diseases. Numerous studies have shown that several cytokines, including tumor necrosis factors (TNFs), interleukins, and interferons (IFNs), are important inflammatory stimulators of VSMCs in vitro and in vivo [3]. These inflammatory stimulators interact with specific receptors and activate signaling cascades, leading to inflammatory responses such as matrix metalloproteinase (MMP) expression; nitric oxide (NO); reactive oxygen species (ROS) production; and subsequent cell growth, adhesion, and migration [3].

Accumulating evidence has indicated that the induction of inducible nitric-oxide synthase (iNOS), a key enzyme for NO biosynthesis, contributes to the process of vascular diseases, such as atherosclerosis [4]. Vascular inflammatory responses induced by pathogens or cytokines are accompanied by the generation of peroxynitrite, a potent and vasotoxic molecule formed through the reaction of NO and superoxide [5]. In addition, a study showed that iNOS contributes to TNF-*α*-induced inflammation and regulates vascular endothelial functions [6]. Previous studies on TNF-*α* have reported a positive correlation through signal transduction pathways that converge at mitogen-activated protein kinases (MAPKs) [7] or nuclear factor-*κ*B (NF-*κ*B) [8]. Conversely, cellular responses to inflammatory stimuli mainly involve the activation of Akt signaling cascades. Akt is involved in the phosphatidylinositol 3-kinase (PI3K)/Akt signaling pathway, which regulates cellular processes, such as cell proliferation, survival, and inflammation [9], and was reported to be essential to TNF-*α*-induced NF-*κ*B activation [10].

**Figure 1:**
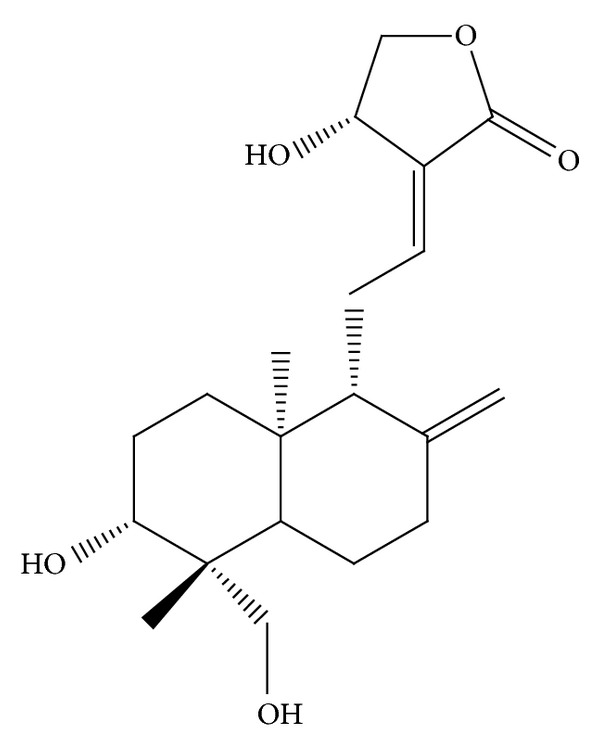
Chemical structure of andrographolide (Andro).

Andrographolide (Figure 1), a novel NF-*κ*B inhibitor, is the most active and critical constituent isolated from the leaves of* Andrographis paniculata* [11].* A. paniculata *has long been used as herbal medicine to prevent and treat upper respiratory tract infections, diarrhea, rheumatoid arthritis, and laryngitis in Asia and Scandinavia [11, 12]. Our previous studies have revealed that andrographolide enhances NF-*κ*B subunit p65 Ser536 dephosphorylation and ROS formation by stimulating neutral sphingomyelinase-mediated ceramide formation in VSMCs [13, 14] and inhibits platelet aggregation by suppressing the p38MAPK/HO^−^-NF-*κ*B-extracellular-signal-regulated-kinase (ERK) 2 cascade [15, 16]. Although andrographolide has exhibited anti-inflammatory activity in various cell types, its anti-inflammatory mechanism in VSMCs remains unclear. In the present study, by considering the pivotal role of VSMC inflammation in the development of atherosclerosis and restenosis [17], we investigated in detail the protective cellular signaling events associated with andrographolide in rat VSMCs stimulated by TNF-*α*, which represented vascular inflammatory conditions.

## 2. Materials and Methods

### 2.1. Materials

Dulbecco's modified Eagle's medium (DMEM), trypsin (0.25%), L-glutamine, penicillin/streptomycin, and fetal bovine serum (FBS) were purchased from Gibco (Gaithersburg, MD, USA). Andrographolide (≥98%), TNF-*α*, LY294002, SP600125, and dimethyl sulfoxide (DMSO) were obtained from Sigma-Aldrich (St. Louis, MO, USA). The anti-iNOS rabbit polyclonal antibody (pAb) and the anti-p65 antibody were purchased from Santa Cruz Biotechnology (Dallas, TX, USA); the anti-*α*-tubulin mouse monoclonal antibody (mAb) was purchased from Thermo Scientific (Waltham, MA, USA); and the anti-phospho-p38 MAPK Thr180/Tyr182 rabbit pAb, anti-p38 MAPK, anti-phospho-p44/p42 extracellular signal-regulated kinase (ERK1/2) Thr202/Tyr204 rabbit pAb, anti-ERK1/2 antibody, anti-phospho-JNK Thr183/Tyr185 rabbit mAb, anti-JNK antibody, anti-phospho-Akt Ser473 rabbit pAb, anti-Akt antibody, anti-phospho-p65 Ser536 rabbit pAb, and anti-I*κ*B*α* antibody were purchased from Cell Signaling (Danvers, MA, USA). A hybond-P polyvinylidene difluoride (PVDF) membrane, an enhanced chemiluminescence (ECL) western blotting detection reagent and analysis system, the horseradish-peroxidase- (HRP-) conjugated donkey anti-rabbit immunoglobulin G (IgG), and the sheep anti-mouse IgG were acquired from Amersham (Buckinghamshire, UK). Andrographolide was dissolved in 0.1% DMSO and stored at 4°C until it was used.

### 2.2. Rat Aortic Smooth Muscle Cell Primary Culture

The male Wistar rats used in this study were purchased from BioLASCO (Taipei, Taiwan). The VSMCs were enzymatically dispersed from the male Wistar rats (250–300 g). Thoracic aortas from the Wistar rats were removed and stripped of the endothelium and adventitia. The VSMCs were obtained using a modification of the combined collagenase and elastase digestion method [18]. These cells were grown in DMEM supplemented with 20 mM HEPES, 10% FBS, 1% penicillin/streptomycin, and 2 mM glutamine at 37°C in a humidified atmosphere of 5% CO_2_. The growth medium was changed every 2-3 d until the cells reached confluence. The growth medium was removed, and the monolayer was rinsed with phosphate-buffered saline (PBS). A trypsin-EDTA solution was added, and the monolayer was incubated at 37°C for 2 min. The culture dishes were observed under a phase-contrast microscope until the cells detached. The cells were removed using 10 mL of DMEM and centrifuged at 900 rpm for 7 min. The pellet was resuspended in DMEM in a culture dish, and cells from Passages 4–8 were used in all experiments. All protocols were approved by the Taipei Medical University Animal Care and Use Committee.

### 2.3. Cell Morphology

**Figure 2:**
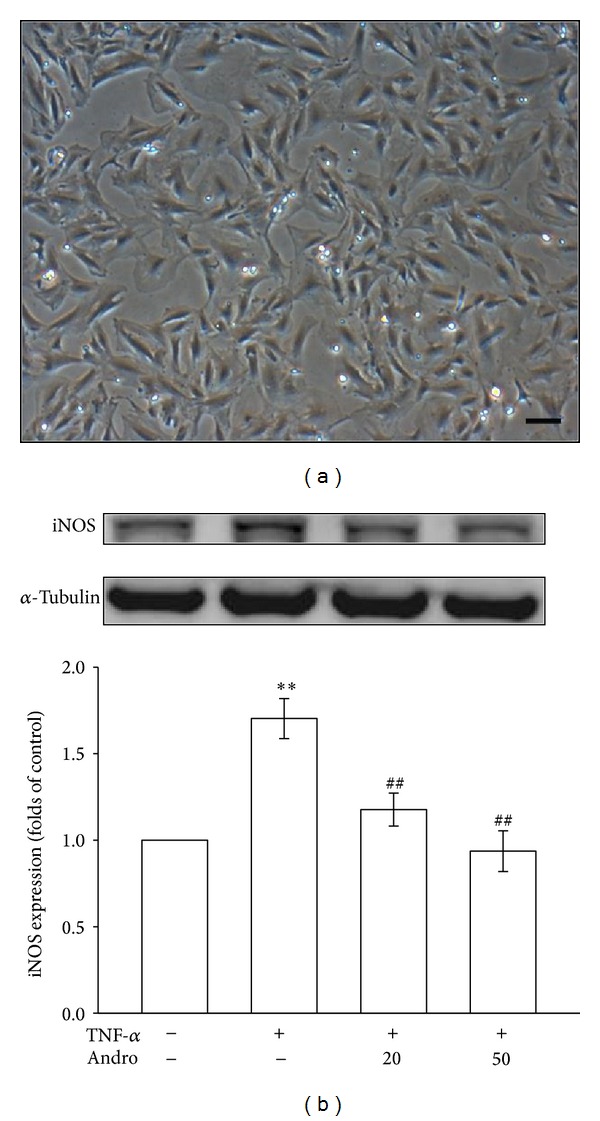
Effects of andrographolide on iNOS expression in TNF-*α*-stimulated VSMCs. (a) Photomicrograph showing the primary cultured rat aortic VSMCs (magnification ×100). (b) The VSMCs were treated with PBS (resting group) or pretreated with andrographolide (20 and 50 *μ*M) or an equal volume of DMSO (solvent control) for 20 min, and TNF-*α* (10 ng/mL) was subsequently added for 24 h. The iNOS protein level was evaluated as described in Section 2.**P < 0.01 compared with the resting group; ^##^ *P* < 0.01 compared with the TNF-*α* group. The data are presented as the mean ± SEM (*n* = 3).

The VSMCs (5 × 10^5^ cells/dish) were seeded in 60-mm dishes and cultured in DMEM containing 10% FBS for 24 h. Cell morphology was evaluated by phase contrast microscopy without preliminary fixation. The primary cultured rat aortic VSMCs exhibited “hills and valleys” pattern (Figure 2(a)), and the expression of *α*-smooth muscle actin was confirmed (data not shown). The micrographs were recorded using a Nikon phase-contrast microscope (Tokyo, Japan).

### 2.4. Immunoblot Analysis

Immunoblot analyses were performed as described previously [18]. Briefly, the VSMCs (5 × 10^5^ cells/dish) were treated as the experimental design. After the experimental period, the proteins were extracted using a lysis buffer. Lysates were centrifuged, the supernatant protein (50 *μ*g) was collected and subjected to sodium dodecyl sulfate-polyacrylamide gel electrophoresis, and the separated proteins were electrophoretically transferred onto 0.45-*μ*m PVDF membranes by using semidry transfer (Bio-Rad, Hercules, CA, USA). The blots were blocked with TBST (10 mM Tris-base, 100 mM NaCl, and 0.01% Tween 20) containing 5% bovine serum albumin for 1 h and then probed with various primary antibodies. The membranes were incubated with HRP-linked anti-mouse IgG or anti-rabbit IgG (diluted 1 : 3000 in TBST) for 1 h. The immunoreactive bands were detected using an ECL system. Bar graphs depict the ratios of quantitative results obtained by scanning the reactive bands and quantifying the optical density by using video densitometry (Bio-Profil; Biolight Windows application Version 2000.01; Vilber Lourmat, France).

### 2.5. Statistical Analysis

The experimental results are expressed as the means ± standard error and are accompanied by the number of observations. Data were assessed using an analysis of variance. If an analysis indicated significant differences among the group means, then each group was compared with the other groups by using the Newman-Keuls method. Values of *P* < 0.05 indicated statistical significance.

## 3. Results

### 3.1. Effects of Andrographolide on the Expression of Inducible Nitric Oxide Synthase in Tumor Necrosis Factor-*α*-Stimulated Vascular Smooth Muscle Cells

We examined whether andrographolide affects the protein level of iNOS, which catalyzes NO formation, in TNF-*α*-stimulated VSMCs. As shown in Figure 2(b), treatment with TNF-*α* increased iNOS expression 1.7 ± 0.1-fold compared with the iNOS expression observed in the control group (*P* < 0.01, *n* = 3). Concentration-dependent inhibition was observed in TNF-*α*-stimulated VSMCs in response to treatment with 20 *μ*M and 50 *μ*M andrographolide; specifically, iNOS expression decreased to 29.4% and 47.1%, respectively.

### 3.2. Effects of Andrographolide on p38 Mitogen-Activated Protein Kinase, Extracellular Signal-Regulated Kinase 1/2, c-Jun N-Terminal Kinase, and Akt Signaling Pathways in Tumor Necrosis Factor-*α*-Stimulated Vascular Smooth Muscle Cells

**Figure 3:**
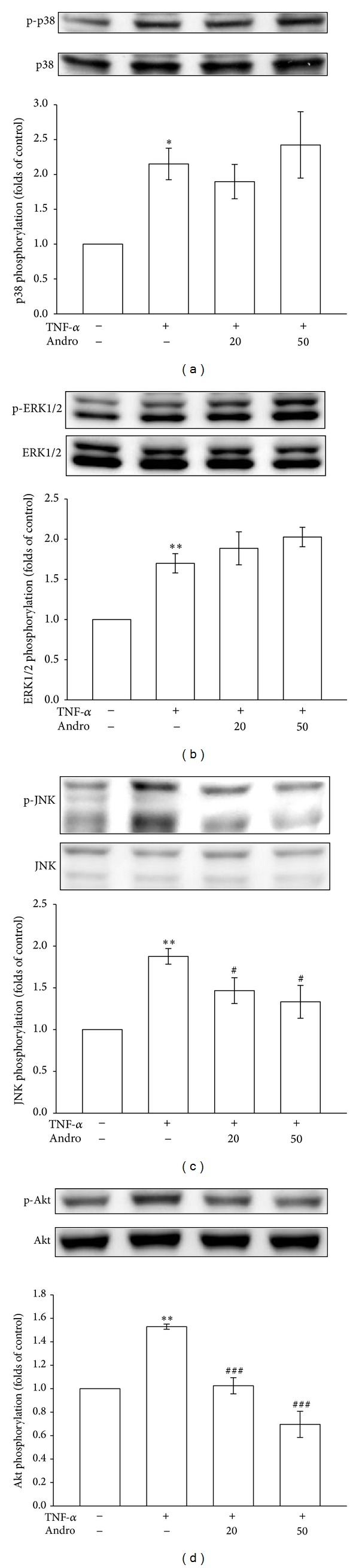
Effects of andrographolide on p38MAPK, ERK1/2, JNK, and Akt signaling pathways in TNF-*α*-stimulated VSMCs. The VSMCs were treated with PBS (resting group) or pretreated with andrographolide (20 and 50 *μ*M) or an equal volume of DMSO (solvent control) for 20 min, and TNF-*α* (10 ng/mL) was subsequently added for 10 min. (a) p38MAPK phosphorylation, (b) ERK1/2 phosphorylation, (c) JNK phosphorylation, and (d) Akt phosphorylation were evaluated as described in Section 2.*P < 0.05 and **P < 0.01 compared with the resting group; ^#^ *P* < 0.05 and ^###^ *P* < 0.001 compared with the TNF-*α* group. The data are presented as the mean ± SEM (*n* = 3).

Vascular inflammation is intensively included in cardiovascular disease. In response to inflammatory stimuli, MAPK phosphorylation increases and subsequently promotes VSMC proliferation and migration [19]. To explore the mechanisms through which andrographolide inhibits TNF-*α*-induced vascular inflammation, we examined the effects of andrographolide on the status of p38MAPK, ERK1/2, and JNK activation in TNF-*α*-stimulated VSMCs. As shown in Figure 3(a), the increase in p38MAPK phosphorylation was 2.2 ± 0.2-fold (*P* < 0.05, *n* = 3) after the VSMCs were exposed to TNF-*α* for 10 min. In addition, 10-min TNF-*α* treatment caused 1.7 ± 0.1-fold and 1.9 ± 0.1-fold increases in ERK1/2 and JNK phosphorylation (*P* < 0.01, *n* = 3) (Figures 3(b) and 3(c)). Pretreating the cells with 20 and 50 *μ*M andrographolide significantly reduced TNF-*α*-induced JNK phosphorylation (Figure 3(c)). However, pretreating the cells with 20 or 50 *μ*M andrographolide did not significantly affect TNF-*α*-induced p38MAPK or ERK1/2 phosphorylation (Figures 3(a) and 3(b)). By contrast, a study reported that Akt is a crucial regulator involved in cell metabolism, cell growth, and vascular inflammation [20].  Figure 3(d) shows that Akt phosphorylation increased 1.5 ± 0.0-fold (*P* < 0.01, *n* = 3) after the VSMCs were exposed to TNF-*α* for 10 min. Pretreating the cells with 20 and 50 *μ*M andrographolide significantly reduced TNF-*α*-induced Akt phosphorylation (Figure 3(d)). These results collectively suggested that andrographolide suppresses vascular inflammation by inhibiting JNK and Akt signaling cascades in TNF-*α*-stimulated VSMCs.

### 3.3. Effects of Andrographolide on I*κ*B*α* Degradation and p65 Phosphorylation in Tumor Necrosis Factor-*α*-Stimulated Vascular Smooth Muscle Cells

**Figure 4:**
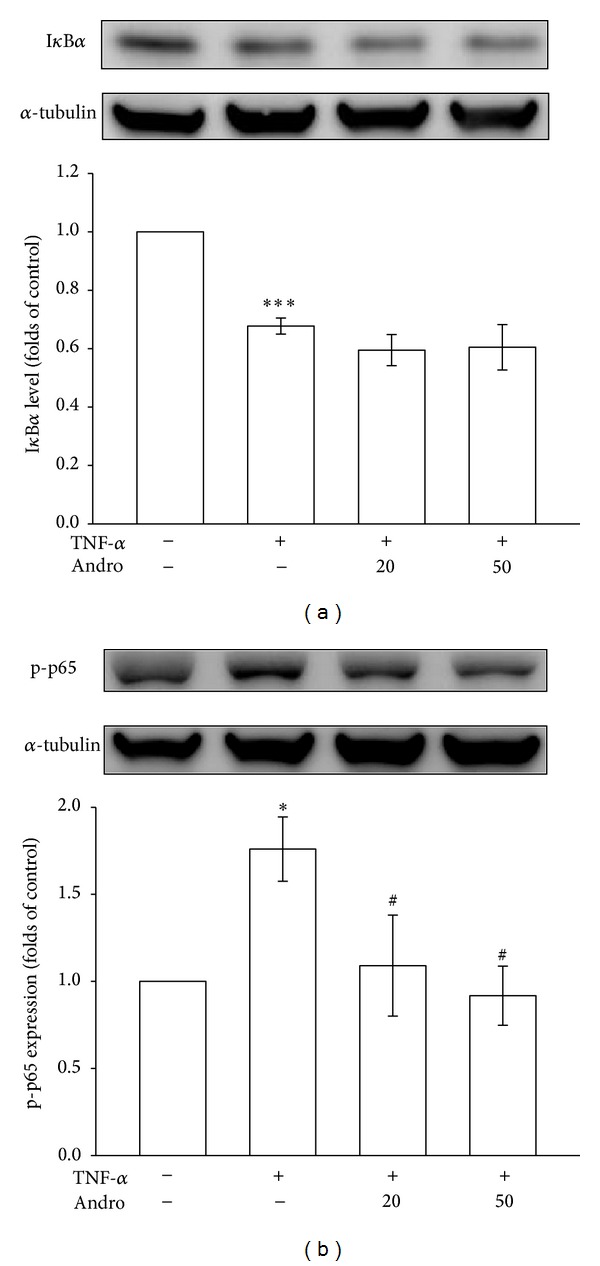
Effects of andrographolide on I*κ*B*α* degradation and p65 activation in TNF-*α*-stimulated VSMCs. The VSMCs were treated with PBS (resting group) or pretreated with andrographolide (20 and 50 *μ*M) or an equal volume of DMSO (solvent control) for 20 min, and TNF-*α* (10 ng/mL) was subsequently added for 30 min. (a) I*κ*B*α* degradation and (b) p65 phosphorylation were evaluated as described in Section 2.*P < 0.05 and ***P < 0.001 compared with the resting group; ^#^ *P* < 0.05 compared with the TNF-*α* group. The data are presented as the mean ± SEM (*n* = 3).

Several studies have observed that NF-*κ*B, a transcription factor, regulates the expression of numerous inflammatory proteins, including iNOS [21]. To clarify the mechanism through which andrographolide inhibits iNOS expression, we evaluated the effect of andrographolide on the level of I*κ*B*α*, a cellular protein that masks the nuclear localization signals of NF-*κ*B and keeps them sequestered in an inactive state [22], in the cytoplasm of TNF-*α*-stimulated VSMCs. However, as shown in Figure 4(a), pretreating the cells with 20 and 50 *μ*M andrographolide did not reverse TNF-*α*-induced I*κ*B*α* degradation. Several studies have indicated that p65 phosphorylation on serine 536 residue mediating its dimerization, DNA binding, and nuclear localization was not associated with or regulated by I*κ*B*α* [23, 24]. As shown in Figure 4(b), pretreating VSMCs with 50 *μ*M andrographolide significantly inhibited (47.7%) TNF-*α*-induced p65 phosphorylation compared with that in TNF-*α*-stimulated VSMCs that were not treated with andrographolide (*P* < 0.05, *n* = 3). These results suggested that p65 phosphorylation rather than I*κ*B*α* degradation is responsible for the andrographolide-mediated inhibition of NF-*κ*B activation in TNF-*α*-stimulated VSMCs.

### 3.4. Andrographolide Suppresses p65 Phosphorylation by Inhibiting the Akt and c-Jun N-Terminal Kinase Signaling Pathways in Tumor Necrosis Factor-*α*-Stimulated Vascular Smooth Muscle Cells

**Figure 5:**
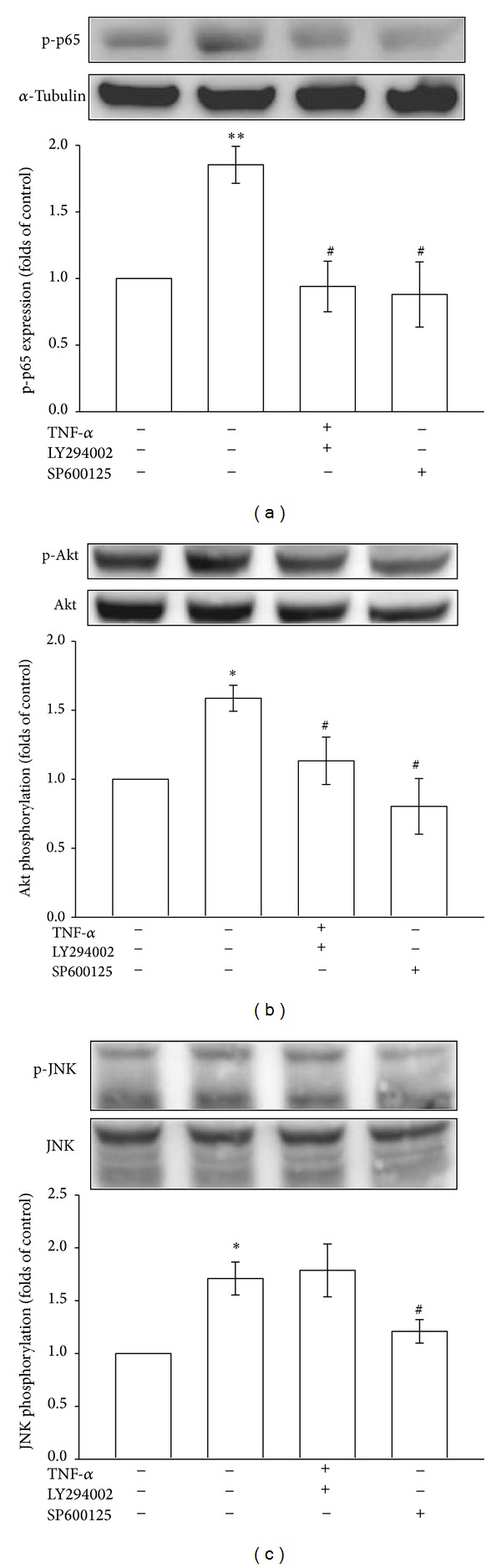
Regulatory effects of various signal inhibitors on p65 activation and Akt and JNK phosphorylation in TNF-*α*-stimulated VSMCs. The VSMCs were treated with PBS (resting group) or pretreated with LY294002 (10 *μ*M), SP600125 (10 *μ*M), or an equal volume of DMSO (solvent control) for 20 min, and TNF-*α* (10 ng/mL) was subsequently added for 10 min ((b) and (c)) or 30 min (a). (a) p65 phosphorylation, (b) Akt phosphorylation, and (c) JNK phosphorylation were evaluated as described in Section 2.*P < 0.05 and **P < 0.01 compared with the resting group; ^#^ *P* < 0.05 compared with the TNF-*α* group. The data are presented as the mean ± SEM (*n* = 3).

To clarify the correlations between Akt, JNK, and andrographolide-induced p65 de-phosphorylation in TNF-*α*-stimulated VSMCs, we used LY294002 (a PI3K/Akt inhibitor) and SP600125 (a JNK inhibitor) to confirm whether Akt or JNK signaling contributes to TNF-*α*-induced p65 phosphorylation in VSMCs. As shown in Figure 5(a), LY294002 at 10 *μ*M significantly inhibited TNF-*α*-induced p65 phosphorylation. Similarly, a JNK inhibitor, SP600125 (10 *μ*M), effectively attenuated TNF-*α*-induced p65 phosphorylation (Figure 5(a)). We further investigated the relationship between Akt and JNK phosphorylation and found that LY294002 (10 *μ*M) and SP600125 (10 *μ*M) obviously diminished TNF-*α*-induced Akt phosphorylation in VSMCs (Figure 5(b)), whereas LY294002 had no significant effects on TNF-*α*-induced JNK phosphorylation except SP600125 (Figure 5(c)). These results collectively suggested that andrographolide suppresses p65 phosphorylation by inhibiting the JNK-Akt signaling cascade in TNF-*α*-stimulated VSMCs.

## 4. Discussion

Our previous study suggested that andrographolide inhibits LPS/IFN-*γ*-induced iNOS and MMP-9 expression in rat VSMCs and revealed that andrographolide reduced neointimal formation in a rat carotid injury model [13]. Recent studies have indicated that andrographolide inhibits TNF-*α*-induced PI3K/Akt phosphorylation and subsequent NF-*κ*B activation in vascular endothelial cells [25, 26]. In addition, in a mouse model of vascular injury, mice lacking functional TNF-*α* developed 14-fold less neointima than wild-type mice did [27]. We hypothesized that the anti-inflammatory effects of andrographolide in the rat model of vascular injury are related to the modulating effects of andrographolide in TNF-*α*-stimulated VSMCs. Therefore, the objective of this study was to examine the effect of andrographolide on signaling molecules involved in TNF-*α*-stimulated VSMCs. During vascular inflammation, TNF-*α* gene transcription was time-dependently upregulated, indicating the active involvement of TNF-*α* in the development of cardiovascular disease [28]. TNF-*α* is a pleiotropic cytokine, and its receptor binding leads to the activation of MAPK, Akt, and NF-*κ*B signaling cascades [29], thereby eliciting a broad spectrum of cellular responses involved in the control of VSMC proliferation, migration, apoptosis, and inflammation. In the present study, to increase the potential for using andrographolide to treat cardiovascular diseases, we showed that andrographolide inhibits TNF-*α*-induced iNOS expression in rat VSMCs.

TNF-*α* induces VSMC inflammation through signal transduction pathways that converge at MAPKs or NF-*κ*B pathways [29]. MAPKs are activated in response to inflammatory and atherogenic stimuli, such as PDGF-BB, TNF-*α*, oxidative stress, hypertension, and balloon injury, and stimulate the expression of several inducible proteins [30]. Furthermore, cellular responses to inflammatory stimuli involve the activation of Akt signaling cascades. The results reported by Chen et al. [26] indicated that andrographolide reduced TNF-*α*-induced Akt phosphorylation in vascular endothelial cells. Whether MAPKs and Akt contribute to the anti-inflammatory property of andrographolide in VSMCs has not been determined. In the present study, we observed that andrographolide suppresses vascular inflammation by inhibiting JNK and Akt signaling cascades, but not p38MAPK and ERK1/2, in TNF-*α*-stimulated VSMCs.

NF-*κ*B activation is securely controlled to ensure a functional host defense and prevent tumorigenesis and hyperinflammation [22]. The NF-*κ*B common form in mainly cell types is the p65/p50 heterodimer, and NF-*κ*B signaling is governed by the IKK complex, which consists of IKK*α*, IKK*β*, IKK*γ*, and the downstream substrate I*κ*B*α*. After stimulation, activated IKK phosphorylates I*κ*B*α*, leading to I*κ*B*α* degradation, enhanced NF-*κ*B nuclear translocation and subsequent transcriptional activation [31]. However, andrographolide did not affect I*κ*B*α* degradation in this study. Sasaki et al. have suggested that p65 phosphorylated on serine 536 is not associated with or regulated by I*κ*B*α*, that it has a distinct set of target genes, and that it may represent a noncanonical NF-*κ*B pathway that is independent of I*κ*B*α* regulation [24]. In this study, we observed that the inhibition of p65 Ser536 phosphorylation may be related to the andrographolide-mediated inhibition of NF-*κ*B in TNF-*α*-stimulated VSMCs.

Based on the data regarding the effects of andrographolide on MAPKs and Akt in the present study, we postulated that JNK and Akt must be inactivated to enable andrographolide to attenuate p65 phosphorylation. The prototype enzyme activated by PI3Ks is protein kinase B (PKB/Akt), a serine-threonine kinase. Three Akt isoforms are known, namely, Akt1, Akt2, and Akt3. Among these isoforms, Akt1 appears to be the enzyme that is the most relevant to cardiovascular functions [32]. Akt1, a crucial vascular effector of PI3K, plays a determinant role in atheroprotection. In double ApoE-Akt1 knockout mice, atherosclerotic lesions in the aorta and coronary vessels are more severe than those in ApoE-knockout controls. Loss of Akt1 in the vessel wall is associated with increased inflammatory signaling [33]. Thus, PI3Kg/Akt1 should be considered a fundamental molecular axis for the pathobiology of atherosclerosis. JNK is an inflammatory and stress-sensitive kinase. Because JNK plays contradictory roles in cell growth and death, the relative activation of these proteins is vital for the inflammatory status of the cell. As crucial upstream regulators in VSMC inflammation, Akt and JNK play key roles in the pathology of atherosclerosis [30, 32]. Studies support the hypothesis that MAPK is required for the activation of several transcription factors, including NF-*κ*B [34]. Bergmann et al. also demonstrated that the inhibitor of p38 MAPK SB203580 abolished TNF-*α*-induced cytokine synthesis and blocked NF-*κ*B-mediated luciferase transactivation [35]. As shown in Figure 5, we observed that treatment with LY294002 (a PI3K/Akt inhibitor) and treatment with SP600125 (a JNK inhibitor) in TNF-*α*-stimulated VSMCs reversed the andrographolide-mediated inhibition of p65 phosphorylation; this observation is consistent with the results of a previous study that indicated that the PI3K/Akt and JNK signaling pathways regulate NF-*κ*B activation [26, 36]. In addition, based on our results, LY294002 and SP600125 diminished Akt phosphorylation, whereas LY294002 had no effects on JNK phosphorylation. Golden et al. have also found that combination of the MAPK Kinase-JNK1 signaling module with Akt represents a crucial stress-activated signalosome that may present protection to sustain cardiac contractility and maintain normal levels of Ca^2+^ [37]. These results may indicate that JNK, as a crucial upstream regulator, plays a key role to regulate the Akt phosphorylation in TNF-*α*-stimulated VSMCs.

**Figure 6:**
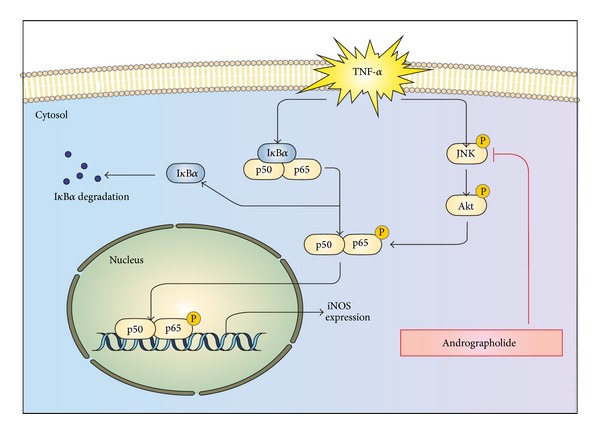
Diagram of the hypothetical inhibitory mechanism of andrographolide-induced effects in TNF-*α*-stimulated VSMCs. TNF-*α* triggers the expression of iNOS through I*κ*B*α*-(I*κ*B*α*-p65) and I*κ*B*α*-independent (JNK-Akt-p65) pathways.

Multiple lines of evidence have suggested that a NO-derived oxidant, peroxynitrite, contributes to inflammatory cardiovascular diseases, such as atherogenesis [5]. Our results indicated that andrographolide significantly diminished iNOS expression in TNF-*α*-stimulated VSMCs by attenuating the Akt and JNK signaling cascade. Moreover, our data suggested that the I*κ*B*α*-independent inhibition of NF-*κ*B activation occurs through the JNK-Akt signaling cascade to regulate the activation of p65 phosphorylation (Figure 6). In conclusion, andrographolide is a potential therapeutic agent that can be applied in treating and preventing inflammatory vascular diseases.
